# Low-Intensity Pulsed Ultrasound Promotes Oligodendrocyte Maturation and Remyelination by Down-regulating the Interleukin-17A/Notch1 Signaling Pathway in Mice with Ischemic Stroke

**DOI:** 10.34133/research.0676

**Published:** 2025-04-25

**Authors:** Jingjing Wang, Yuxiao Gao, Bin Wang, Cong Zhang, Yi Yuan, Renhao Xu, Hui Ji, Xiangjian Zhang

**Affiliations:** ^1^ Department of Neurology, Second Hospital of Hebei Medical University, Shijiazhuang, Hebei 050000, China.; ^2^ Hebei Key Laboratory of Vascular Homeostasis and Hebei Collaborative Innovation Center for Cardio-cerebrovascular Disease, Shijiazhuang, Hebei 050000, China.; ^3^ Key Laboratory of Clinical Neurology (Hebei Medical University), Ministry of Education, Shijiazhuang, Hebei 050000, China.; ^4^ School of Electrical Engineering, Yanshan University, Qinhuangdao 066004, China.; ^5^ Key Laboratory of Intelligent Rehabilitation and Neuromodulation of Hebei Province, Yanshan University, Qinhuangdao 066004, China.

## Abstract

Increasing evidence indicates that oligodendrocyte (OL) numbers and myelin as a dynamic cellular compartment perform a key role in the maintenance of neuronal function. Inhibiting white matter (WM) demyelination or promoting remyelination has garnered interest for its potential therapeutic strategy against ischemic stroke. Our previous work has shown that low-intensity pulsed ultrasound (LIPUS) could improve stroke recovery. However, it is unclear whether LIPUS can maintain WM integrity early after stroke or promote late WM repair. This study evaluated the efficacy of LIPUS on WM repair and long-term neurologic recovery after stroke. Male adult C57BL/6 mice underwent a focal cerebral ischemia model and were randomized to receive ultrasound stimulation (30 min once daily for 14 days). The effect of LIPUS on sensorimotor function was assessed by modified neurological severity score, rotarod test, grip strength test, and gait analysis up to 28 days after stroke. We found that ischemic stroke-induced WM damage was severe on day 7 and partially recovered on day 28. LIPUS prevented neuronal and oligodendrocyte progenitor cell (OPC) death during the acute phase of stroke (d7), protected WM integrity, and reduced brain atrophy and tissue damage during the recovery phase (d28). To further confirm the effect of LIPUS on remyelination, we assessed the proliferation and differentiation of OPCs. We found that LIPUS did not increase the number of OPCs (PDGFRα^+^ or NG2^+^), but markedly increased the number of newly produced mature OLs (APC^+^) and myelin protein levels. Mechanistically, LIPUS may promote OL maturation and remyelination by down-regulating the interleukin-17A/Notch1 signaling pathway. In summary, LIPUS can protect OLs and neurons early after stroke and promote long-term WM repair and functional recovery. LIPUS will be a viable strategy for the treatment of ischemic stroke in the future.

## Introduction

Cerebral white matter (WM) constitutes roughly 50% of the total volume of the human brain [1] and consists mainly of myelin, axonal fibers, and myelin-producing oligodendrocytes (OLs). WM injury is a critical part of brain damage after stroke, with a pathologic process that includes death of oligodendrocyte progenitor cells (OPCs) and myelin-producing mature OLs, loss of myelin, and a following axonal degeneration. The severity of cerebral WM injury is markedly associated with post-stroke motor-sensory dysfunction and cognitive decline [2]. Therefore, attenuating cerebral WM damage and promoting cerebral WM repair may contribute to the long-term prognosis of stroke. However, current studies have focused on strategies to protect neurons and promote collateral circulation, largely ignoring the protective role of WM. It is therefore urgent to find treatments that can reduce the death of OLs and promote myelin repair, thereby improving long-term neurological deficits after stroke.

OPCs comprise approximately 5% of all cells in the adult mouse brain [3] and remain capable of proliferation, migration, and differentiation [4]. When OPCs are in proliferation, chondroitin sulfate proteoglycan neuron-glia antigen 2 (NG2) and platelet-derived growth factor receptor (α-subunit, PDGFR-α) are activated, both of which are also often recognized as markers of OPCs [5]. Down-regulation of PDGFR-α and NG2 marks the initiation of OPCs differentiation into OLs. The stages of OLs can be categorized into 2 phases: immature OLs (or known as premyelinating OLs) and mature OLs (myelinating OLs). Immature OLs first express O4, followed by Gpr17, Enpp6, Bcas1, or Tcf7l2. Myelin antigens such as myelin basic protein (MBP), proteolipid protein (PLP), myelin oligodendrocyte glycoprotein (MOG), and 2′,3′-cyclic nucleotide 3′-phosphodiesterase (CNPase) are then markers for mature OLs [5]. At present, there is a growing consensus that the migratory capacity of OPCs and their quantity are not the sole primary constraints for WM repair [6]. Another very critical limiting factor is the lack of an intrinsic or extrinsic environment that supports the differentiation of OPCs to OLs, preventing OPCs recruited to the site of injury from effectively differentiating into mature OLs [7–9]. Existing studies have shown that signaling pathways such as Notch, Wnt, and protein kinase B/mechanistic target of rapamycin (AKT/mTOR) are involved in regulating the differentiation of OPCs to OLs and myelin regeneration [10].

Transcranial ultrasound stimulation (TUS) is an innovative method of neuromodulation that has emerged in recent times. TUS has been studied in ischemic stroke, dementia, mood disorders, and neurodegenerative diseases like Parkinson’s disease and amyotrophic lateral sclerosis. Low-intensity pulsed ultrasound (LIPUS), as a form of TUS, has been widely studied for its reversible, noninvasive, high spatial resolution, and high depth of stimulation in regulating neuronal activity and promoting neuronal function recovery after stroke. There is a close connection between neurons and myelin, and the recovery of neuronal function cannot be separated from the role of myelin. Studies have shown that long-term myelin deficiency leads to neuronal death and dysfunction after stroke [11]. Other studies have also confirmed that neuromodulation is an effective tool not only for restoring neural circuits but also for inducing myelin formation [12–14]. Yang et al. [15] showed that LIPUS accelerated myelin regeneration in multiple sclerosis (MS) mice by inhibiting glial cell activation, enhancing the density of mature OLs, and promoting brain-derived neurotrophic factor (BDNF) production. Huang et al. [16] found that LIPUS increased myelin content in the hippocampus and corpus callosum of rats with vascular dementia, which they suggested may be partially attributable to increased BDNF levels. The above studies strongly point to the fact that neuromodulation may have a palliative effect on myelin loss, representing a new modality for treating WM injury with excellent prospects in the future. However, the exact mechanism of LIPUS is largely unknown, which is highly likely to hinder its development and application. Therefore, we established an ischemic stroke model and first observed the effect of LIPUS on WM integrity and myelin regeneration to clarify its efficacy in post-stroke WM injury. Then, we obtained differentially expressed genes using RNA sequencing (RNA-seq) and verified them to try to illustrate the mechanism of action of LIPUS in restoring WM integrity.

## Results

### LIPUS improves long-term histologic and functional prognosis after ischemic stroke

**Fig. 1.**
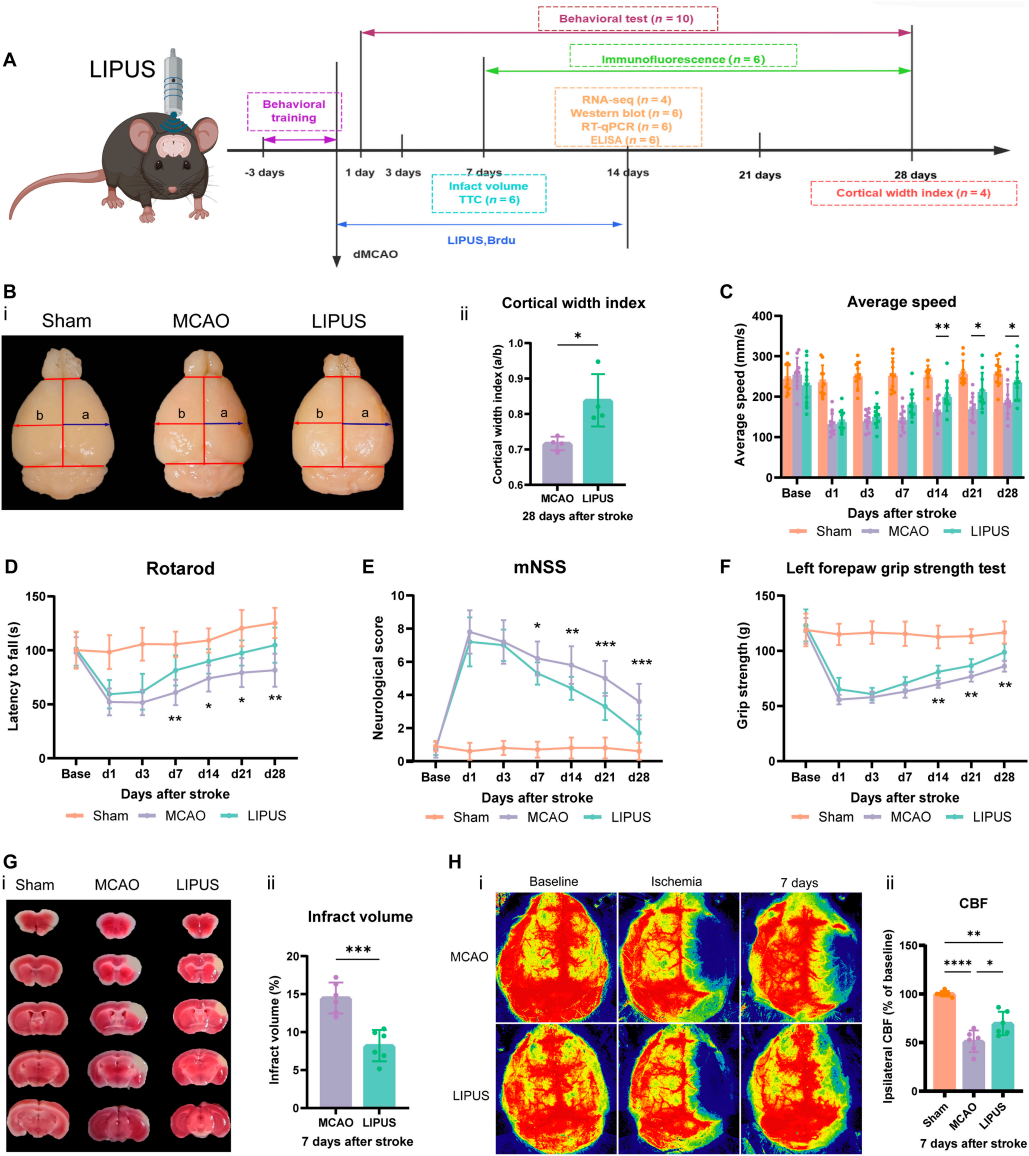
LIPUS improves long-term histologic and functional prognosis after ischemic stroke. (A) Design of the experimental program for this study. (B) Representative images (i) and quantitative analysis plots (ii) of cortical width index on day 28 after stroke, *n* = 4 mice/group. (C to F) Quantitative analysis of mean velocity (C), rotarod test (D), neurological score (E), and left forepaw strength (F) before (baseline) and after dMCAO (days 1, 3, 7, 14, 21, and 28), *n* = 10 mice/group. (G) Representative images of TTC staining (i) and quantitative analysis of infarct volume (ii), *n* = 6 mice/group. (H) Representative images of CBF at baseline, at the time of ischemia, and on day 7 after dMCAO (i), and quantification of cerebral blood flow on postoperative day 7 (ii), *n* = 6 mice/group. The above data are expressed as mean ± SD. The data in (C) to (F) were subjected to repeated-measures ANOVA, while the analysis of (B) and (G) involved an unpaired 2-tailed Student’s *t* test. Additionally, for (H), a one-way ANOVA was conducted followed by Tukey’s multiple comparisons test. *P < 0.05, **P < 0.01, ***P < 0.001,****P < 0.0001.

The experimental protocol is shown in Fig. 1A. To investigate the long-term prognostic impact of LIPUS on stroke, we assessed atrophy volume by cortical width index (CWI) on day 28 after stroke. Studies typically use CWI as a measurement of cortical expansion to define the long-term effects of treatment after ischemia [17,18]. The results showed that the LIPUS intervention group reduced brain atrophy volume compared with the distal middle cerebral artery occlusion (dMCAO) group (Fig. 1B). To investigate the effects of LIPUS on neurological function, we performed behavioral tests on cerebral infarction model mice through a range of behavioral assessments, consisting of modified neurological severity score (mNSS), rotarod test, grip strength test, and gait analysis (Fig. 1C to F). The results showed that LIPUS promoted the recovery of neurological deficits in the middle and late stages after stroke. The LIPUS intervention also showed partial improvement in the early stroke phase (d7 postoperatively). Compared to the dMCAO group, the LIPUS group exhibited a longer stay on the rotarod test as well as a lower mNSS score. Given that the value of CWI depends not only on cortical expansion in the recovery phase but also on infarct volume in the acute phase, we further enumerated infarct volume and cerebral blood flow (CBF) recovery in the acute phase of post-stroke. The results indicated a reduction in the size of the infarct and an improvement in CBF recovery in the LIPUS group when compared to the dMCAO group(Fig. 1G and H).

### LIPUS improves cerebral WM integrity in late ischemic stroke

**Fig. 2.**
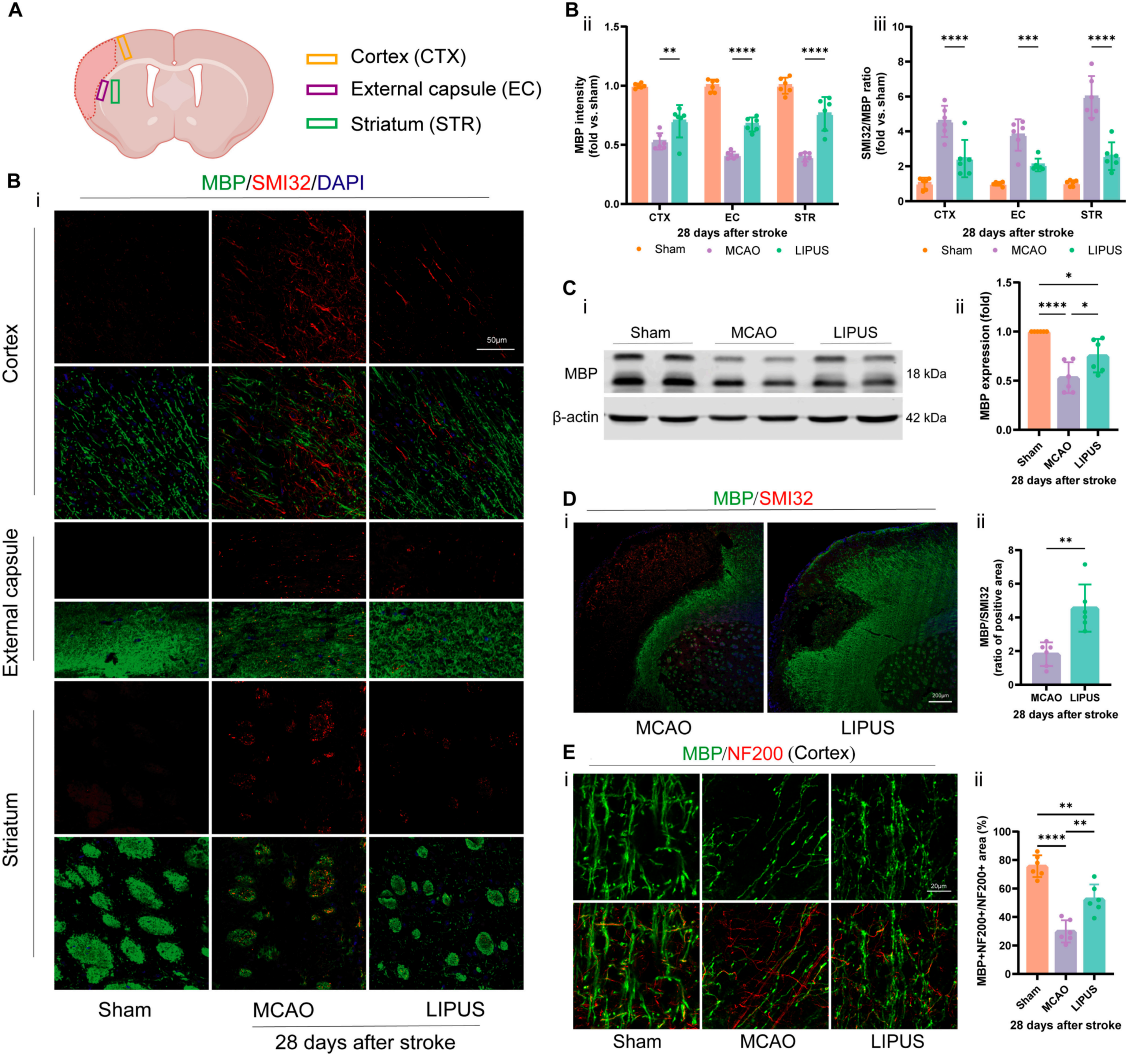
LIPUS improves cerebral WM integrity on day 28 after stroke. (A) Schematic of the localization of the peri-infarct cortex (CTX), external capsule (EC), and striatum (STR). (B) Double-labeled immunofluorescence staining of MBP (green) and SMI32 (red) in 3 brain regions, CTX, EC, and STR, on day 28 after dMCAO (i). Quantification of MBP immunoreactivity (ii) and the SMI32/MBP fluorescence intensity ratio (iii) are presented as the ratio of fold change compared to the Sham control, scale bar = 50 μm, *n* = 6 mice/group. (C) Representative images depicting that the Western blot analysis of MBPs was obtained for each group (i). The expression levels of the target proteins were normalized relative to β-actin (ii). (D) Representative immunostaining of MBP and SMI32 on day 28 after dMCAO (i), scale bar = 200 μm; (ii) quantification of the ratio of MBP to SMI32 staining-positive areas in ipsilateral injured brain tissue. Data were normalized to stained areas in the contralateral hemisphere, *n* = 6 mice/group. (E) Representative images of myelin coverage (i), calculated MBP+ myelin coverage area along NF200+ nerve fibers (ii), scale bar = 20 μm, *n* = 6 mice/group. Data are presented as means ± SD. Statistical analysis for (B) was conducted using 2-way ANOVA and then Tukey’s multiple comparisons test, while (C) and (E) were analyzed using one-way ANOVA and then Tukey’s multiple comparisons test. Analysis for (D) involved unpaired 2-tailed Student’s *t* test. *P < 0.05, **P < 0.01, ***P < 0.001,****P < 0.0001.

Cerebral WM injury is an important component of ischemic stroke progression. Therefore, we further evaluated the therapeutic effect of LIPUS on cerebral WM injury after cerebral ischemia. WM injury markers were assessed in 3 WM-containing brain regions (cortex, striatum, and external capsule) around the focus of infarction on day 28 after dMCAO (Fig. 2A). Cerebral ischemia negatively affects the integrity of WM, as indicated by a reduction in the signal of MBP, which serves as a marker for assessing myelin integrity. Additionally, there is an elevation in the signal of SMI32, a marker used to detect axonal injury, observed across all regions. Compared to the mice in the dMCAO group, the LIPUS group mice exhibited notable enhancements in WM injury repair, as demonstrated by an increase in MBP intensity and a decrease in the SMI32/MBP ratio in the WM-enriched areas (Fig. 2B). Meanwhile, LIPUS-treated mice had significantly higher MBP and SMI32 staining area ratios on day 28 after stroke compared with dMCAO group mice (Fig. 2D). In addition, we also found that myelin coverage was higher after LIPUS treatment by calculating the myelin coverage of MBP^+^ along NF200^+^ (200 kDa neurofilament, an axon marker) fibers (Fig. 2E). Consistent with the immunofluorescence results, MBP expression levels in the peri-infarct brain regions were higher in the LIPUS group mice compared with the dMCAO group mice in Western blot analysis (Fig. 2C). All of the above indicated the promoting effect of LIPUS on myelin regeneration after stroke.

### LIPUS reduces early neuron and OPC death after ischemic stroke

**Fig. 3.**
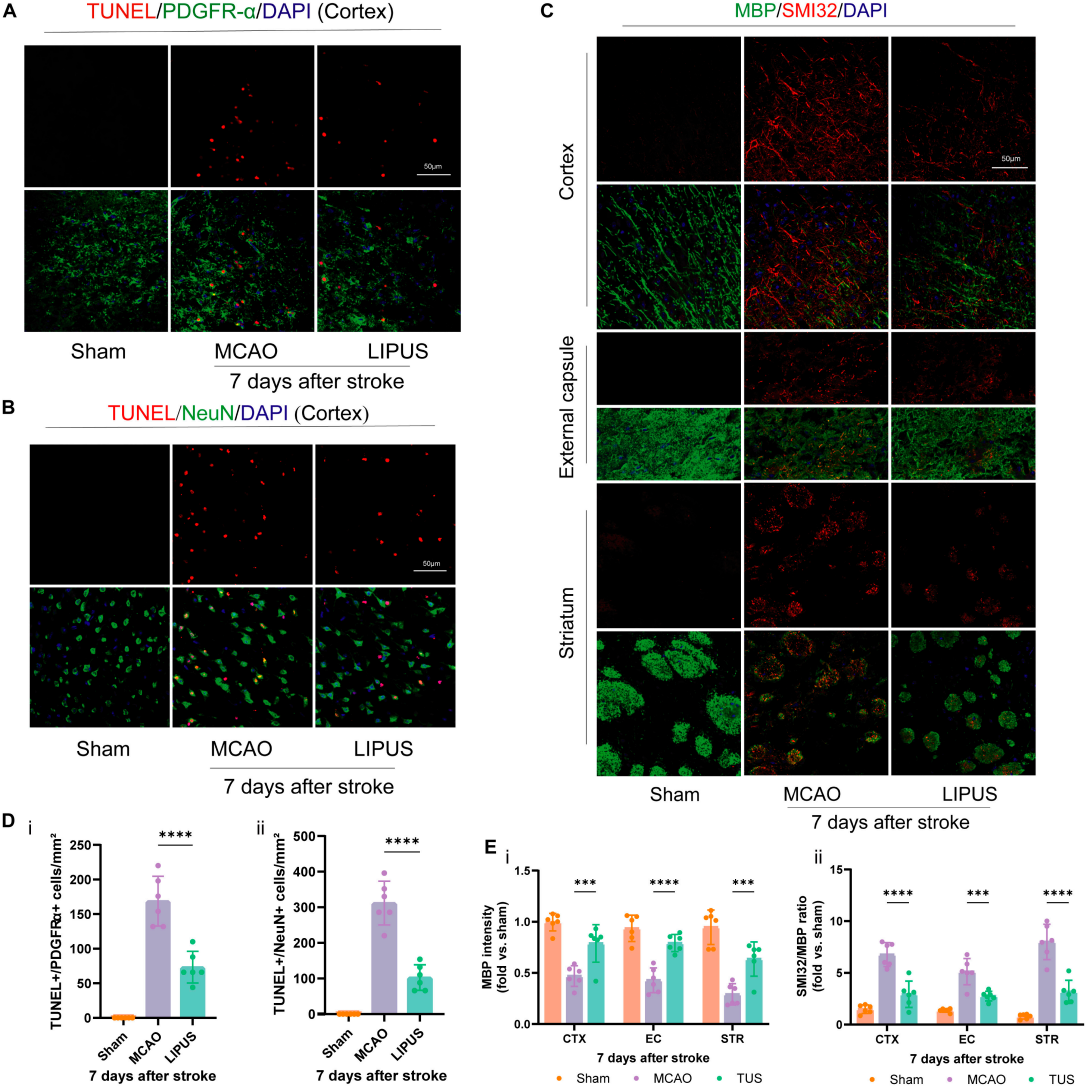
LIPUS reduces early death of neurons and OPCs and improves WM integrity after ischemic stroke. Representative images (A) and quantitative analysis (D, i) of TUNEL (a marker of cell death) and PDGFR-α (a marker of OPC) immunostaining in the peri-infarct cortex on day 7 after stroke. Representative images (B) and quantitative analysis (D, ii) of TUNEL and NeuN (a marker of neurons) immunostaining in the peri-infarct cortex on day 7 after stroke. Representative images (C) of double-labeled immunofluorescence staining for MBP and SMI32 in 3 brain regions (CTX, EC, and STR) on day 7 after dMCAO. Quantification of MBP immunoreactivity (E, i) and SMI32/MBP fluorescence intensity ratio (E, ii) is indicated as fold change relative to Sham control. Scale bar = 50 μm, *n* = 6 mice/group. Data are presented as means ± SD. (A) was analyzed using a 2-way ANOVA followed by Tukey’s post hoc test, while (B) and (C) were assessed using a one-way ANOVA followed by Tukey’s post hoc analysis. ***P < 0.001, ****P < 0.0001.

As shown in the above results, LIPUS reduced infarct volume in the acute phase (d7) and also improved WM integrity and functional recovery in the long term (d28). Timely salvage of brain cells, including neurons and OLs, early after stroke onset may be beneficial for long-term functional recovery. Here, we assessed whether LIPUS reduced the death of neurons and OPCs. Double immunostaining for NeuN^+^/TUNEL^+^ (Fig. 3B) and PDGFR-α^+^/TUNEL^+^ (Fig. 3A) showed that the number of dead neurons (NeuN^+^TUNEL^+^) and OPCs (PDGFR-α^+^TUNEL^+^) was reduced in the cerebral cortex of the LIPUS-treated group of mice compared with that of control mice on day 7 after dMCAO. In addition, through the SMI32/MBP immunofluorescence intensity ratio, we verified the early demyelination damage caused by ischemia on cerebral WM and the ameliorating effect of LIPUS on early myelin loss (Fig. 3C).

### LIPUS promotes the differentiation of OPCs after ischemic stroke

**Fig. 4.**
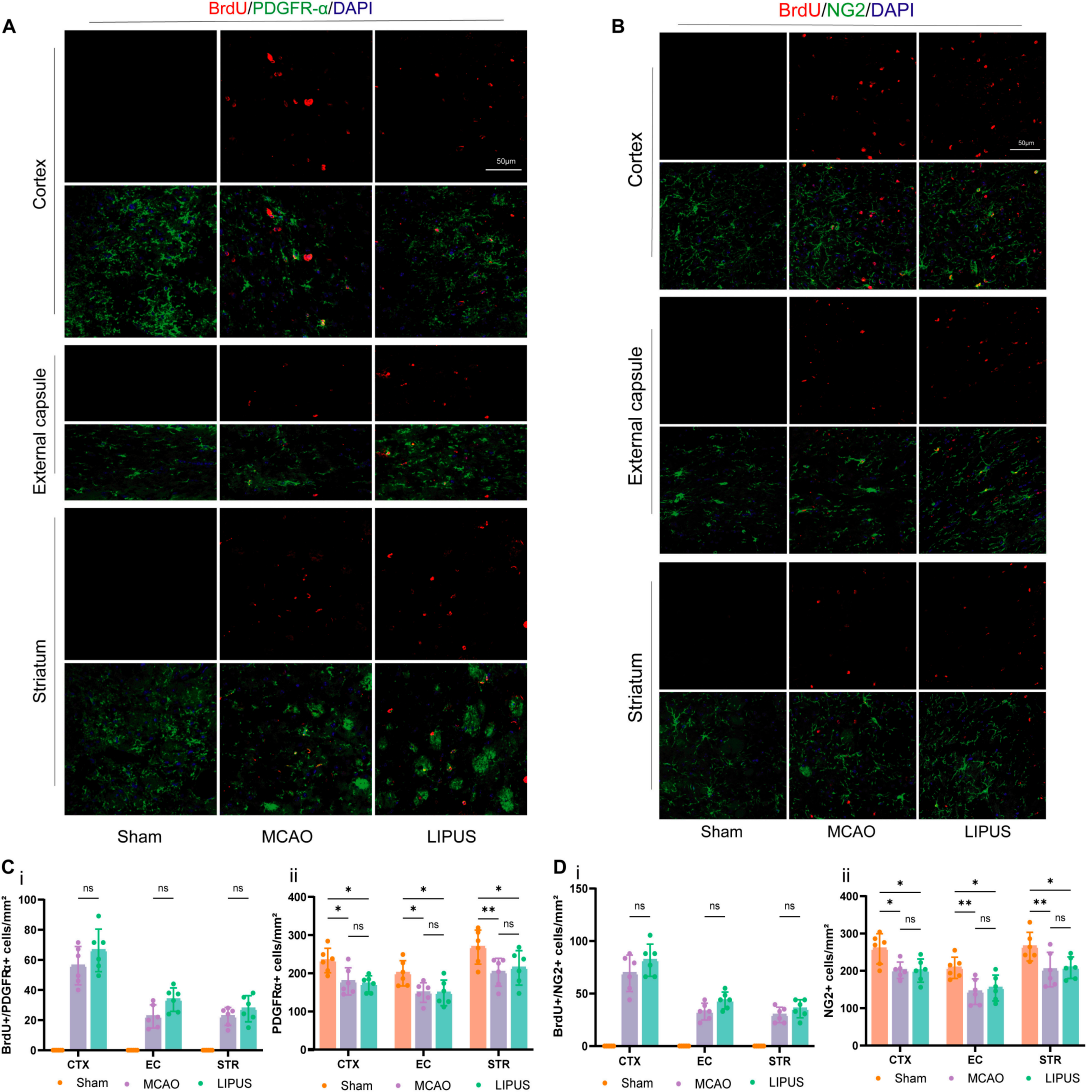
LIPUS has a nonsignificant influence on OPC proliferation after ischemic stroke. (A) and (B) depict immunostaining results of BrdU (a marker for cell proliferation, shown in red) along with 2 OPC markers PDGFR-α and NG2 (shown in green), observed in 3 brain regions on day 14 post-stroke. (C) and (D) are quantitative analyses of (A) and (B), counting the number of Brdu^+^PDGFR-α (C, i), PDGFR-α^+^ cells (C, ii), Brdu^+^NG2^+^ cells (D, i), and NG2^+^ cells (D, ii). Scale bar = 50 μm, *n* = 6 mice/group. Data are presented as means ± SD. The statistical analysis of (C) and (D) involved conducting a 2-way ANOVA and subsequently applying Tukey’s multiple comparisons test. *P < 0.05, **P < 0.01. ns = no statistical difference.

Oligodendrogenesis is critical for WM repair after stroke. We therefore analyzed the proliferation and differentiation of OPCs on day 14 after stroke. We measured the proliferation of OPCs after dMCAO by detecting OPCs labeled with 5-bromo-2′-deoxyuridine (BrdU). Both PDGFR-α and NG2 were markers for OPCs. The results showed that BrdU^+^PDGFR-α^+^ cells were significantly increased in the cortex, external capsule, and striatum around the infarct foci in mice in the dMCAO group, suggesting that ischemic injury initiated the proliferation of OPCs (Fig. 4A and C). Nevertheless, LIPUS treatment did not significantly enhance ischemia-induced proliferation of OPCs, and there was no statistical difference between the 2 groups. We obtained similar results using NG2 to label OPCs again (Fig. 4B and D). This suggests that LIPUS has little effect on the proliferation of OPCs in vivo.

**Fig. 5.**
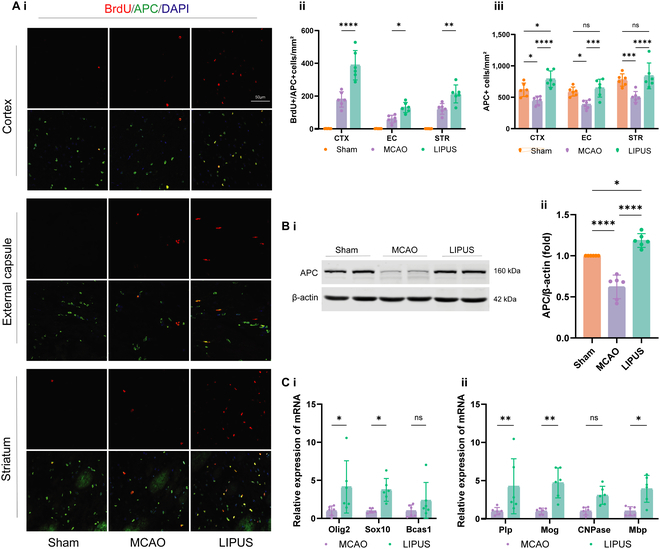
LIPUS promotes the production of newly matured OLs after ischemic stroke. (A) Quantification of newborn OLs in the peri-infarct region (CTX, EC, and STR) on day 14 after stroke. (i) Representative images showing the colocalization of APC (green) and BrdU (red) through double immunofluorescence staining, (ii) statistical analysis depicting the quantity of BrdU^+^APC^+^ cells, and (iii) statistical analysis illustrating the overall number of APC^+^ cells. Scale bar = 50 μm, *n* = 6 mice/group. (B) Representative images depicting that the Western blot analysis of APC protein expression levels in the peri-infarct brain region was obtained at postoperative day 14 (i). The expression levels of the target proteins were normalized relative to β-actin (ii), *n* = 6 mice/group. (C, i) RT-qPCR was performed to detect the expression of OL differentiation-related transcription factors (Olig2, Sox10, and Bcas1) and (C, ii) myelin-related genes (Plp, Mog, CNPase, and Mbp). The experiment was repeated 3 to 5 times. The obtained results were first standardized against glyceraldehyde-3-phosphate dehydrogenase (GAPDH) and subsequently normalized with respect to the control group (MCAO group), *n* = 6 mice/group. Data are presented as means ± SD. Statistical analysis was conducted using 2-way ANOVA followed by Tukey’s multiple comparisons test for (A) and (C), and one-way ANOVA followed by Tukey’s multiple comparisons test for (B). *P < 0.05, **P < 0.01, ***P < 0.001, ****P < 0.0001, ns = no statistical difference.

The formation of new myelin depends more on the ability to differentiate into new mature OLs than on the number of OPCs. Therefore, we further evaluated the differentiation of OPCs in 3 brain regions around the infarct. BrdU^+^APC^+^ double immunofluorescence staining was used to label the newly formed mature OLs. The results showed that there were almost no BrdU^+^APC^+^ cells in the normal group (Sham), and a small number of OLs cells were visible in the dMCAO group, whereas the number of BrdU^+^APC^+^ cells was significantly increased after LIPUS treatment (Fig. 5A). Western blot analysis further verified that LIPUS promoted the expression level of APC in stroke mice (Fig. 5B). In addition, the differentiation and maturation of OLs are regulated by a series of transcription factors. We conducted additional analysis using quantitative polymerase chain reaction (qPCR) to examine the expression of various transcription factors associated with myelin formation. The reverse transcription (RT)-qPCR results demonstrated that LIPUS treatment increased the expression levels of pro-differentiation-related genes (Olig2 and Sox10), as well as a significant up-regulation of myelin-related genes such as PLP, MBP, and MOG in mice with cerebral ischemia (Fig. 5C). The above data suggest that LIPUS has a protective effect on WM injury after cerebral ischemia by promoting the differentiation of OPCs.

### LIPUS down-regulates IL-17A expression and Notch1 activation after ischemic stroke

**Fig. 6.**
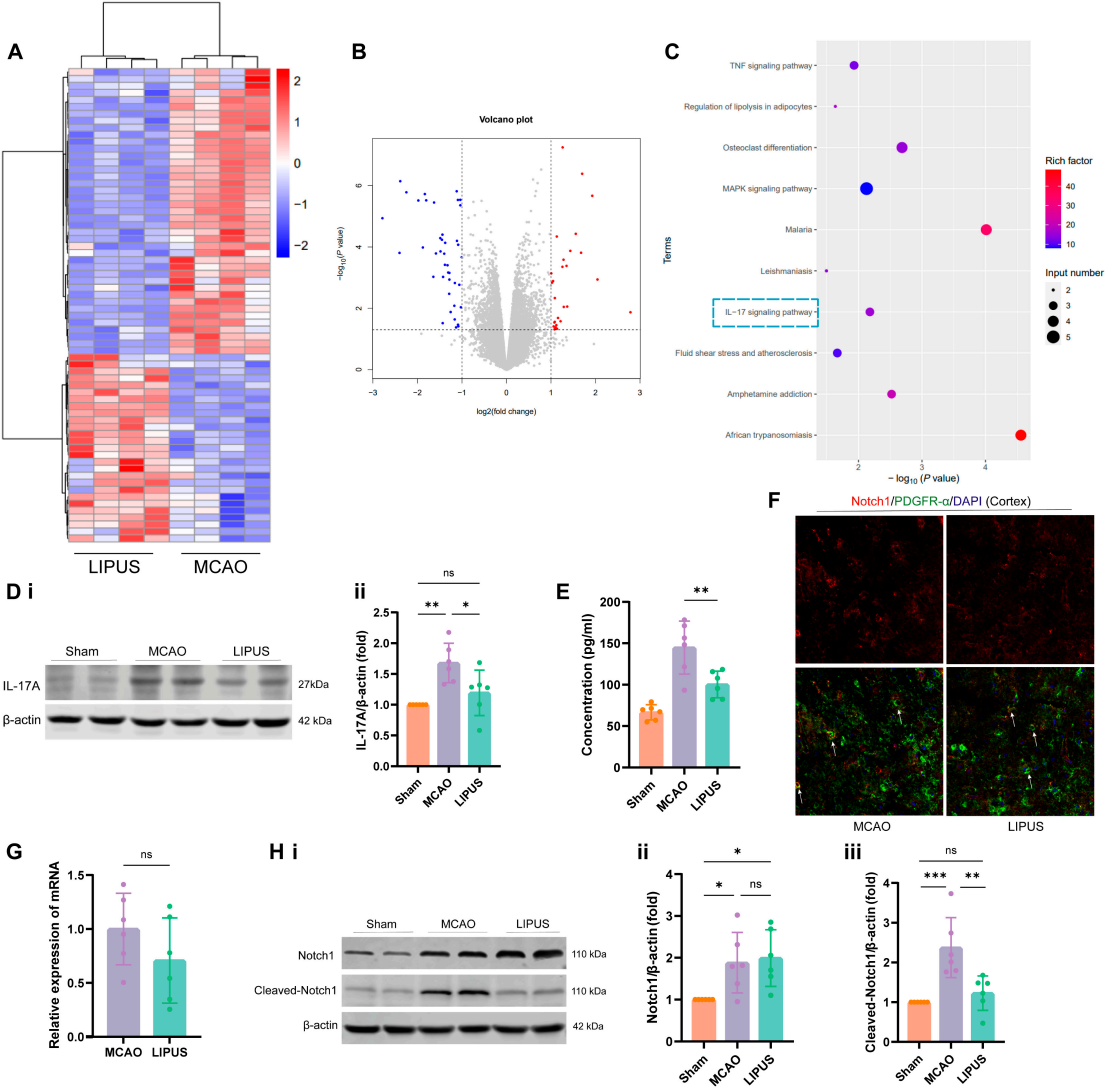
LIPUS down-regulates IL-17A expression and Notch1 activation after ischemic stroke. (A to C) RNA-seq analysis was performed on ischemic penumbra brain tissue from dMCAO nonintervention and LIPUS-treated mice at postoperative day 14, *n* = 4 mice/group. (A) The results of hierarchical cluster analysis for differential genes between the 2 groups were displayed using a heatmap. (B) A volcano plot was employed to visualize the changes in gene expression (up-regulation in red and down-regulation in blue) detected by RNA-seq between LIPUS-treated mice and nonintervention MCAO mice. (C) KEGG pathway analysis revealed alterations in several important pathways within the ischemic penumbral brain tissue following LIPUS intervention. (D) Differences in IL-17A protein levels within the peri-infarct brain regions of Sham, MCAO, and LIPUS group mice are shown with representative images in (i) and quantitative plots in (i). β-Actin serves as a loading control. *n* = 6 mice/group. (E) Expression of IL-17A in brain tissue homogenates from 3 groups of mice by ELISA. *n* = 6 mice/group. (F) Representative maps of double immunofluorescence staining for Notch1 (red) and PDGFR-α (green) in peri-infarct cortex. (G) RT-qPCR detection of Notch1 expression. Experiments were repeated 3 to 5 times. *n* = 6 mice/group. (H) Western blot analysis of protein levels of Notch1 and its activated state (Cleaved-Notch1) in semi-dark band brain tissues. (i) Representative images and (ii and iii) quantitative statistical plots. *n* = 6 mice/group. Data are reported as means ± SD. Statistical analysis for comparisons between groups (D, E, and H) was conducted using one-way ANOVA followed by Tukey’s multiple comparisons test, while an unpaired 2-tailed Student’s *t* test was employed for the analysis of group (G). *P < 0.05, **P < 0.01, * *P < 0.001, ns = no statistical difference.

To assess the influence of LIPUS on the expression profile of genes in brain tissues after ischemic stroke and to search for genes related to the promotion of OPC differentiation, we collected ischemic penumbra brain tissues of each group of stroke mice for RNA-seq experiments on day 14 postoperatively (the period of obvious proliferation and differentiation). Gene expression patterns were found to be modified in the brain tissues of each group of mice (Fig. 6A to C). Compared with the Sham group, 407 genes were increased and 12 genes were decreased in expression in the dMCAO group. In the brain tissues of mice in the LIPUS group, there was an increase in the expression of 27 genes and a decrease in the expression of 41 genes compared to mice in the dMCAO group (Fig. 6A and B). Kyoto Encyclopedia of Genes and Genomes (KEGG) pathway analyses showed that key genes were enriched in 10 signaling pathways (Fig. 6C). We selected the IL-17 signaling pathway of interest, which is linked to myelin formation. We examined the expression level of IL-17A by enzyme-linked immunosorbent assay (ELISA) and Western blot (Fig. 6D and E) and confirmed that LIPUS reduced the IL-17A content of brain tissues in stroke mice.

To further identify the downstream targets of LIPUS affecting the differentiation of OPCs through IL-17A, we chose Notch1 for validation. It is widely accepted that Notch1 signaling is a negative regulator of OPC differentiation, which is confirmed in many published studies. Another study showed that IL-17 stimulation induced Notch1 activation in OPCs and promoted Th17-mediated demyelinating disease [19]. Similar to previous reports, we first corroborated Notch1 expression in OPCs by double immunofluorescence staining of PDGFR-α with Notch1 (Fig. 6F). Next, we examined Notch1 changes at the gene level in mouse penumbra brain tissues by qPCR and found that although LIPUS tended to reduce its transcript levels, the difference was not statistically significant (Fig. 6G). We proceeded to further analyze the Notch1 protein expression level by Western blot technique and again there was no difference between the 2 groups. We then focused on the activation of Notch1. Upon activation of Notch1, the Notch1 intracellular structural domain (NICD), which is attached to the inner side of the cell membrane, was cleaved, translocated to the nucleus, and promoted the transcription of Notch target genes. Compared with the dMCAO group, LIPUS significantly reduced Cleaved Notch1 (NICD) cleavage and decreased activation of Notch1 signaling (Fig. 6H).

### LIPUS promotes differentiation of OPCs and improves cerebral WM integrity after ischemic stroke by inhibiting the IL-17A/Notch1 pathway

**Fig. 7.**
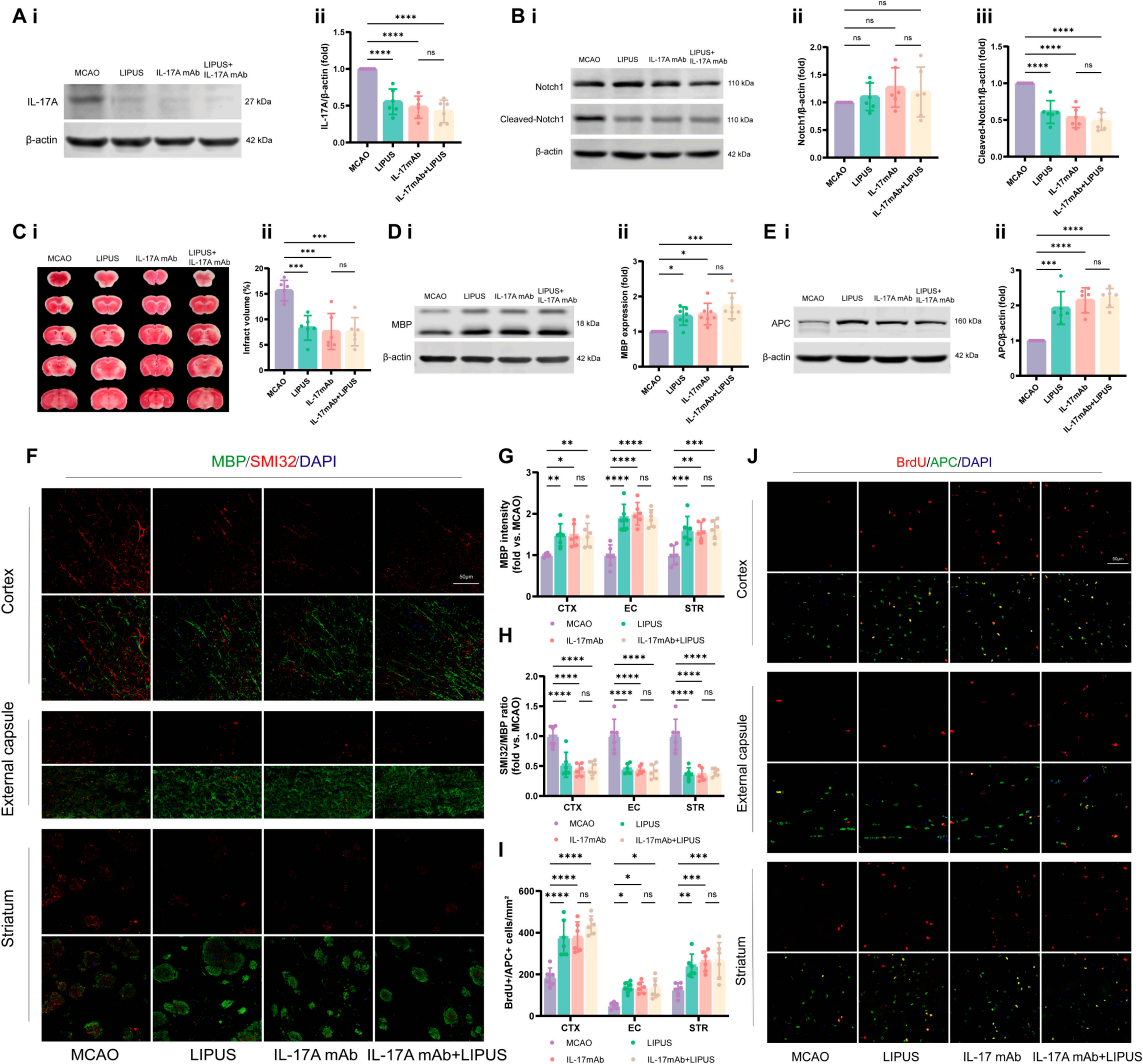
LIPUS promotes differentiation of OPCs and improves cerebral WM integrity after ischemic stroke by inhibiting the IL-17A/Notch1 pathway. (A) Western blot analysis of the protein levels of IL-17A in 4 groups of penumbra brain tissues, with representative images (i) and quantitative analysis plots (ii), *n* = 6 mice/group. (B) Western blot analysis of the protein levels of Notch1 and Cleaved-Notch1 in 4 groups of penumbra brain tissues, with representative images (i) and quantitative analysis plots of Notch1 (ii) and Cleaved-Notch1 (iii), *n* = 6 mice/group. (C) Representative photographs of TTC staining (i) and quantitative analysis of infarct volume (ii), *n* = 6 mice/group. (D) Representative images depicting that the Western blot analysis of MBP was obtained for each group (i). The expression levels of the target proteins were normalized relative to β-actin (ii), *n* = 6 mice/group. (E) Representative images depicting that the Western blot analysis of APC was obtained for each group (i). The expression levels of the target proteins were normalized relative to β-actin (ii), *n* = 6 mice/group. (F) Double-labeled immunofluorescence staining of MBP (green) and SMI32 (red) in 3 brain regions, CTX, EC, and STR, scale bar = 50μm, *n* = 6 mice/group. (G) is the quantification of MBP immunoreactivity in (F), and (H) is the SMI32/MBP intensity ratio in (F). (J) Representative images showing the colocalization of APC (green) and BrdU (red) through double immunofluorescence staining, scale bar = 50μm, *n* = 6 mice/group. (I) is a quantitative map of Brdu+APC+ cells in (J). Data are presented as means ± SD, (A) to (E) were performed by one-way ANOVA followed by Tukey’s multiple comparisons test. (G) to (I) were performed by a 2-way ANOVA followed by Tukey’s multiple comparisons test.*P < 0.05, **P < 0.01, ***P < 0.001, ****P < 0.0001, ns = no statistical difference.

To further verify whether IL-17A/Notch1 signaling is involved in the process of LIPUS promoting the generation of mature OLs in ischemic stroke mice, we added IL-17A monoclonal antibody (IL-17A mAb) to inhibit IL-17A signaling. First, we verified the inhibition of IL-17A expression in the brain of ischemic stroke mice by injection of IL-17A mAb by Western blot analysis (Fig. 7A). Further, we tested whether blocking IL-17A played an inhibitory role in its downstream Notch1 activation. The results showed that the expression of IL-17A and Cleaved Notch1 was decreased in the LIPUS, IL-17A mAb, and LIPUS+IL-17A mAb groups compared to the dMCAO group. Among the comparisons between the IL-17A mAb and LIPUS+IL-17A mAb groups, no statistically different results were observed (Fig. 7A and B).

Next, we evaluated the critical role of IL-17A/Notch1 involvement in LIPUS for neuroprotection after MCAO. The use of LIPUS or IL-17A mAb alone or a combination of both resulted in a significant reduction in cerebral infarct size on day 7 postoperatively (Fig. 7C). On day 28 postoperatively, all 3 groups showed an increase in MBP fluorescence intensity as well as a decrease in the SMI32/MBP ratio in the peri-infarct cortex, external capsule, and striatum (Fig. 7F to H). Western blot similarly confirmed the increased MBP expression levels, suggesting a significant improvement in cerebral WM integrity (Fig. 7D). On day 14 after surgery, the number of BrdU^+^/APC^+^ colabeled mature OLs was significantly increased in all 3 groups compared with the dMCAO group (Fig. 7I and J), and the protein expression level of APC was similarly increased (Fig. 7E). There remained no statistical difference between the IL-17A mAb group and the LIPUS+IL-17A mAb group in terms of the expression levels of APC and MBP. Taken together, these results suggest that LIPUS promotes the differentiation of OPCs and improves the integrity of cerebral WM after stroke mainly through IL-17A/Notch1 signaling.

## Discussion

Our current study demonstrates that LIPUS ameliorates WM injury after stroke and improves the long-term prognosis of stroke. We demonstrate that LIPUS inhibits IL-17A/Notch1 signaling, which promotes the differentiation of OPCs and the repair of WM damage.

Myelin synthesized by OLs forms myelin structures that wrap around axons and play a crucial role in signaling in functional neural networks [20]. Cerebral WM consists mainly of bundles of myelin sheaths and axons. Ischemic stroke causes significant brain WM damage, leading to long-term sensorimotor impairment and cognitive dysfunction [21,22]. Consistent with previous studies, our results showed severe demyelination of the peri-infarct cortex, external capsule, and striatum in the acute phase (d7) after ischemic stroke and remission of demyelination with partial compensatory myelin recovery occurring in the late phase (d28). The application of LIPUS markedly reduced infarct volume in the acute phase, promoted blood flow recovery, and markedly improved poststroke WM injury, thereby promoting sensorimotor recovery. Unfortunately, however, we did not assess cognitive function.

OL death-induced demyelination is one of the mechanisms leading to WM injury. After the onset of cerebral ischemia, OLs and their precursor cells, because of their sensitivity to ischemia-induced oxidative stress and excitotoxicity [22,23], lead to massive OL death, which induces demyelination and WM injury. On the other hand, cerebral ischemia also triggers compensatory cerebral WM repair to promote neurological recovery [24]. An important mechanism involved in WM repair is remyelination [25]. After demyelination of the CNS, OPCs are recruited to the area of injury, differentiate into mature OLs and produce new myelin sheaths, a natural regenerative process known as remyelination [26]. Although ischemia induces compensatory remyelination of brain tissue, unfortunately, this compensatory regeneration is limited. Studies have shown that OPCs are evenly dispersed across the central nervous system, accounting for approximately 5% of the total population of brain cells in adult mice [3,27]. When cerebral ischemia occurs, OPCs in the ipsilateral peri-infarct brain regions and subventricular zone (SVZ) regions undergo proliferation and recruitment to the infarct zone [28]. Although the typical role of OPCs is to produce myelinated OLs, extensive research has been conducted in recent years on the non-myelin-forming functions of OPCs [3,29]. There is growing evidence that OPCs play an equally important role in promoting functional recovery of neuronal and vascular endothelial cells[11,30]. OPCs transform into functionally distinct groups depending on intrinsic mechanisms and extrinsic environment, which reduces their ability to differentiate into myelin [9,31–33]. While OPCs may exhibit active proliferation following a stroke, the differentiation of OPCs into mature OLs is rare, leading to limited restoration of WM. Therefore, interventions that promote differentiation of OPCs may be more helpful in promoting myelin formation, WM rehabilitation, and neurological recuperation in stroke patients. In our study, we observed compensatory remyelination after stroke. Proliferation of OPCs in the surrounding areas of the infarct including external capsule, cortex, and striatum was evident on day 14 after dMCAO, and a small number of mature OLs were also seen. LIPUS intervention did not markedly affect the OPCs’ proliferation but markedly enhanced the maturation of the OLs. The OPCs’ differentiation demands the function of differentiation-promoting transcription factors like Olig1, Olig2, Sox10, Nkx2.6, and Bcas1 [8,34–36]. We chose Olig2, Sox10, and Bcas1 for validation and found elevated Olig2 and Sox10. In addition, we also concurrently focused on the initial damage in early stroke (d7). It has been shown that LIPUS markedly reduces neuronal apoptosis in ischemic brain tissue, prevents cell viability decline, and ameliorates neuronal injury [37,38]. In our results, LIPUS not only reduces ischemia-induced neuronal death in the early phase but also reduces apoptosis in OPCs, which we believe plays a role in attenuating early demyelination injury and promoting late WM repair.

TUS, a noninvasive neuromodulation technique, has undergone a decade-long development [39]. Apart from TUS, other noninvasive techniques for neuromodulation encompass transcranial magnetic stimulation, transcranial direct current stimulation, and transcranial alternating current stimulation. These techniques have been used clinically to promote the recovery of neurological function in patients with central nervous system diseases. Compared to the latter, TUS is superior in targeting the cerebral cortex and deeper regions of the brain with a spatial resolution of up to a few cubic millimeters. Zhang et al. [40] even demonstrated a model for dissolving intravascular blood clots through high-speed vortex ultrasound, which may hold new promise for future stroke treatments. The LIPUS we studied (intensity typically <100 W/cm^2^) acts through mechanical effects (activation of the mechanosensitive ion channels TRPP1/2, TRPC1, TRPA1, and Piezo1, among others, as described in the review [41]) and at the same time does not produce heating effects that cause damage to the target area. Studies have shown that LIPUS activates or inhibits the electrical activity of brain tissues through its advantages of reversible, noninvasive, high spatial resolution, and deep stimulation in a way that improves functional deficits after stroke. Firstly, the anti-inflammatory effect of LIPUS should be mentioned. Wang et al. [42] used LIPUS (500 kHz) to stimulate the ischemic hemisphere of MCAO mice, which induced microglial cell polarization toward M2 and up-regulated IL-10/IL-10R signaling, resulting in an improvement of the neurological prognosis and a reduction of the volume of brain atrophy on days 7 to 14 after the operation. Qi et al. [43] show that LIPUS (500 kHz) improves blood flow and synaptic behavior on day 7 after MCAO by up-regulating HMGB1 and down-regulating CAMK2N1 in specific astrocyte subpopulations. Deng et al. [44] found that LIPUS (500 kHz) stimulation reduced the volume of cerebral edema in MCAO mice, improved neurological function, increased CBF by 20%, and reduced the secretion of inflammatory factors tumor necrosis factor-α (TNF-α) and matrix metalloproteinase-9 in the ischemic brain. This is consistent with our findings that LIPUS reduced the inflammatory response on day 14 after MCAO, and RNA-seq analysis showed a down-regulation of multiple inflammatory signaling pathways including TNF, MAPK, and IL-17. This is similar to the results of a study on LIPUS-stimulated amyotrophic lateral sclerosis mice. Their RNA-sequencing analysis showed that LIPUS markedly reduced the expression of TNF-α pathway, IL-17 pathway, and nuclear factor kappa B pathway components associated with neuroinflammation in the motor cortex [45]. We chose IL-17 signaling as our primary study subject for validation and confirmed the down-regulation of IL-17 by ELISA and Western blot at multiple levels. In addition, the role of LIPUS in improving neurological recovery in stroke patients cannot be ignored. Noninvasive neuromodulation is currently at the forefront of ischemic stroke rehabilitation. LIPUS stimulation of neurons in the cortex or deep brain nuclei (e.g., cerebellar dentate nucleus [46], thalamus [47–49], and amygdala [50]) can help to restore their neurological functions. LIPUS also promotes the regeneration of cerebral blood vessels after a stroke, helping to restore blood supply to the damaged area [43,51]. A growing number of studies have confirmed the positive role of noninvasive neuromodulation techniques in promoting functional recovery of the central nervous system, but the specific mechanisms are still unclear. Studies on the pro-myelinogenic effects of LIPUS have mainly focused on MS model mice and have not been studied in the MCAO model. Our study reveals for the first time that LIPUS plays a reparative role in ischemia-induced WM injury by promoting the differentiation of OPCs and reveals the possible mechanisms.

The basic parameters of LIPUS include intensity, fundamental frequency (FF), pulse repetition frequency (PRF), stimulation duration (SD), duty cycle (DC), tone burst duration (TBD), spatial peak time-averaged intensity (Ispta), and spatial peak pulse-averaged intensity (Isppa). Differences in the settings of ultrasound parameters may lead to variations in their mechanisms of action (cavitation, thermal, and mechanical effects). Several mechanisms may occur individually or simultaneously, leading to different neuromodulatory effects [52]. Currently, the parameter settings of most studies on LIPUS are centered on a fundamental frequency of 0.25 to 1 MHz, an Ispta of 3 to 30 W/cm^2^, a PRF of 1 kHz or less, and an SD of about a few hundred milliseconds [53]. The middle cerebral artery (MCA) is most often affected during stroke. Depending on the location of the MCA occlusion, the severity of ischemia is different and the ischemic brain area is different. Part of the damage involves only the cortex, and part of the damage involves both the cortex and deep subcortical brain structures. LIPUS can modulate neural activity (excitation or inhibition) in the cortex and subcortical brain regions, including primary somatosensory cortex (S1), secondary somatosensory cortex (S2), primary motor cortex (M1), primary visual cortex (V1), thalamus, amygdala, and dentate nucleus of the cerebellum, among others, through the skull [41]. Chen et al. [54] examined the transmission efficiency of ultrasound through the skull at different fundamental frequencies and found that frequencies of 150 kHz could achieve transmission efficiencies of up to 20% to 30% of the sound intensity, whereas frequencies of 1,000 kHz could only achieve transmission efficiencies of less than 5% of the sound intensity. In addition, the transmission efficiency at 500 kHz is higher than at 350 kHz, while relatively low transmission efficiencies occur at frequencies greater than 750 kHz. Therefore, in most animal and human studies, 500 kHz is set more often. Parameters other than FF, such as PRF, DC, and SD, also play important roles in neuromodulation. Both Qi et al.’s and Deng et al.’s studies used transient middle cerebral artery occlusion (tMCAO) for 90 min to induce a focal ischemic brain injury model. The FF (500 kHz) and SD (300 ms) were the same in both studies, but the PRF was different, 500 and 1,000 Hz, respectively [43,44]. Their studies all support that LIPUS improves CBF in ischemic brain regions and promotes neurological recovery. Our study used a lower PRF (1 Hz) and a shorter SD (50 ms), which also improved CBF. The difference is that our study used the dMCAO model with only cortical damage, whereas they used the tMCAO model with more severe damage. The difference in stroke models resulted in different ischemic brain regions and different stroke severity. Clinical stroke patients present with more complex ischemic manifestations depending on the occluded vessel, which may range from punctate infarcts due to small-vessel occlusion to large infarcts due to large-artery occlusion. Whether the ultrasound parameters should be adjusted according to the location or severity of the infarction may be an interesting topic in the clinical application of LIPUS.

The IL-17 family comprises 6 cytokines that share a similar structural composition, including IL-17A to IL-17F. IL-17A has been most extensively studied as the first family member to be discovered. We still know very little about IL-17B to IL-17F; thus, for the most part, IL-17A is considered synonymous with the IL-17 family (commonly known as IL-17). What is known by researchers is the involvement of IL-17A in promoting inflammation in autoimmune disorders. In addition, most evidence also suggests that an elevated presence of IL-17A within the brain is associated with its pathogenic effects. IL-17A promotes neurodegeneration and neuroinflammatory responses in MS and ischemic stroke [55–57]. Based on existing studies, IL-17 inhibits OPC proliferation and differentiation by acting on the voltage-gated KC (Kv) channel 1.3 (Kv1.3) [58], Act1 [59], and Notch1 [19] targets of OPCs. There are many signaling pathways regarding the regulation of remyelination [10], including Notch, Wnt, RXR (retinoid X receptor gamma), AKT/PIP3/mTOR, LINGO1 (leucine-rich repeat and Ig domain containing 1), and Nrf2 (nuclear factor erythroid 2-related factor 2) signaling pathways. The majority of current research supports Notch1 as a negative regulator of remyelination. Notch signaling relies on the binding of ligands to extracellular structural domains, leading to the release of active Notch fragments (NICDs) from the cell. After NICDs are released, they enter the nucleus, bind to DNA-binding proteins, and assemble transcriptional complexes. The expression of the Notch1 receptor is observed in OPCs and their differentiation is regulated through the Notch1 signaling pathway [60]. Activation of Notch1 signaling inhibits differentiation of OPCs, resulting in defects in remyelination [19,60]. Zhang’s team successfully developed a mouse model called Olig1Cre: Notch1^12f/12f^, where they specifically deactivated Notch1 in the OL spectrum. As a result of this selective deactivation, the mice exhibited enhanced repair of demyelination damage in the corpus callosum [61]. A study by Wang et al. reported that IL-17 stimulation induced Notch1 activation in OPCs. They suggested that, mechanistically, IL-17R expressed on the cell membrane of OPCs promotes the cleavage of NICD by interacting with Notch1, which also acts as a transmembrane protein. IL-17A-induced activation of Notch1 leads to the interaction of the IL-17R junction Act1 with NICD1, and subsequent translocation of the Act1–NICD1 complex into the nucleus, initiating the activation of genes associated with inflammation and promoting the activation of genes related to cell proliferation in OPCs. Both inhibit the normal differentiation program of OPCs into mature OLs [19].

We chose IL-17A/Notch1 as the study target for validation. Many studies have shown that providing a low-inflammatory environment for OL formation is critical to promote cerebral WM repair after stroke [62]. Our RNA-seq results showed that differential genes were enriched in the MAPK, TNF, and IL-17 signaling pathways, suggesting that LIPUS improves the inflammatory state after stroke. We first selected extracellular signal-regulated kinase (ERK) and p-ERK (a classical branch of the MAPK pathway) for Western blot validation but did not find significant differences in protein levels. Subsequently, we reviewed the literature and found that the IL-17 family, which has been gradually emphasized in recent years, is closely related to myelin regeneration [19,58,63]. Therefore, we chose IL-17A for ELISA and Western blot validation and clarified its decreased protein level in brain tissue. However, unfortunately, we did not count the number of major cell populations (Th17 cells) secreting IL-17A, the ratio of anti-inflammatory/pro-inflammatory cells, and whether they affected apoptosis in early OPCs, which will be the focus of our next work. After determining the decline of IL-17A, we further searched for downstream targets of its action on OPCs to find direct evidence for its promotion of OPC differentiation. By reviewing the literature, we selected Notch1 for validation. The results showed that there was no statistically significant difference in the transcript and protein levels of Notch1, but NICD was markedly reduced in the LIPUS group. Further, we blocked IL-17A by injection of IL-17A mAb and found down-regulation of NICD expression, enhancement of OPCs differentiation, and improvement of brain WM integrity in MCAO mice. MCAO mice with both IL-17A mAb and LIPUS applied did not differ markedly from those with only IL-17A in the changes of the above indexes. Overall, the above experiments suggest that LIPUS promotes OPC differentiation into mature myelin-forming OLs and ameliorates WM damage at least to some extent through IL-17A/Notch1 signaling.

Our study has some shortcomings at the same time. Firstly, we chose ischemic penumbra tissue for transcriptome sequencing and did not isolate OPCs or OLs for sequencing, making the results less specific and affecting the validity of the results. Secondly, we did not deeply investigate the cause of IL-17A decline caused by LIPUS, and we did not elucidate the direct target of LIPUS, which will be the goal of our work for a long time to come. Thirdly, we have not perfected the in vitro experiments, which, like the problems mentioned in the first point, will lead to a partial loss of precision in our study. However, considering ultrasound stimulation as a physical stimulation mode, it is greatly affected by the medium in which it is propagated. Therefore, we did not proceed with the in vitro experiments after fully considering their necessity but rather completed our study with in vivo experiments that were as perfect as possible.

In conclusion, LIPUS improves long-term functional recovery by ameliorating the inflammatory state after stroke and inhibiting IL-17A/Notch1 signaling to promote WM repair, which provides a promising novel therapeutic approach for stroke and other demyelinating diseases.

## Materials and Methods

### Animals

Male C57BL/6 mice were obtained from Vital River Laboratory Animal Technology (China) Co. and were of specific pathogen-free (SPF) grade. These mice weighed 20 to 24 g and were aged 8 to 12 weeks. They were housed in an SPF-grade animal facility with strict control over ambient temperature (22 ± 3 °C) and humidity (60% ± 5%). A daily light–dark cycle of 12 h each was maintained, while the mice had unrestricted access to food and water. After acclimatization, random allocation into control and experimental groups was performed for subsequent experiments. All animal experimental protocols followed the guidelines set by the Animal Care and Use Committee of the Second Hospital of Hebei Medical University (Permit No. HMUSHC-130318), as well as the National Institutes of Health *Guide for the Care and Use of Laboratory Animals* (NIH Publication No. 80-23, revised in 1996).

### Model

Distal cerebral ischemia (dMCAO) was induced in adult male mice by a permanent blockade of the right common carotid artery (CCA) and the right middle distal cerebral artery (MCA). Mice were first anesthetized with isoflurane, and a skin incision was made slightly to the right of the midline of the neck, followed by dissection of the right CCA and permanent ligation. Next, a second skin incision was created in the line connecting the right ear to the eye to expose the skull. A hole about 2 to 3 mm in diameter was made in the right skull with a high-speed dental drill to reveal the distal part of the right MCA, which was occluded with a microbipolar electrocoagulator. Intraoperatively, a heating pad was used to control the temperature of the right temporalis muscle and rectus at about 37.0 ± 0.5 °C. We detected regional CBF in the right cortex of mice using the 2-dimensional laser speckle technique. We deemed the model to be effective if the CBF dropped by 30% compared to the baseline level; otherwise, it was excluded. Additionally, mice were excluded in case of mortality during surgery or occurrence of complications like cerebral hemorrhage or subarachnoid hemorrhage.

### Low-intensity ultrasound stimulation protocol

Our focused ultrasound transducer consists of 3 parts: (a) a signal generator (AFG3022C, Tektronix, USA) [64], which is responsible for generating sinusoidal signals; (b) a power amplifier (E&I 240L, ENI Inc., USA) [64], which is responsible for amplifying the electrical signals emanating from the generator; and (c) a focused ultrasound probe (V301-SU, focus, diameter: 25.4 mm, radius of curvature: 40 mm, Olympus, USA) and a conical collimator (3D-printed, resin material, filled with ultrasound coupling fluid) [64], which were placed at the stimulation site for ultrasound stimulation. Based on our previous studies [64–66], the ultrasound parameters determined for this study were: center frequency = 500 kHz, pulse repetition frequency = 1 Hz, duty cycle = 10%, and stimulation duration = 50 ms. The ultrasound pressure was 0.38 MPa. The Isppa was 4.86 W/cm^2^ and the Ispta was 0.48 W/cm^2^.

Mice were anesthetized using inhalation of isoflurane gas at concentrations between 1.0% and 1.5% under voluntary respiration conditions. We shaved the tops of the mice’s heads and fixed them on a customized stand to keep them stable. Each mouse received 30 min of LIPUS treatment at the same time each day for 14 days.

### Drug treatments

To investigate the impact of LIPUS on the improvement of neurological function following MCAO, we divided stroke mice into 4 groups: (a) IgG isotype control group: 100 μg of mouse IgG1 kappa isotype control (BE0083, BioXcell, USA) was injected intravenously. (b) Ultrasound intervention group: ultrasound stimulation was given. (c) Anti-IL-17 antibody group: intravenous injection of 100 μg of anti-IL-17A antibody derived from mice (BE0173, BioXcell, USA) was administered 1 day after MCAO. (d) Ultrasound + anti-IL-17 antibody group: both ultrasound treatment and intravenous injection of anti-IL-17A antibody were given.

### Behavioral assessment

Four different approaches were employed to evaluate the mice’s neurological function: mNSS, accelerated Rotarod test, grip strength test, and gait analysis. For the latter 3 testing methods, mice were required to be trained on each task 3 to 5 days before the stroke for them to reach a stable performance. We then recorded the results of these mice undergoing these tests on days 1, 3, 7, 14, 21, and 28 after dMCAO. All tests were performed by experimenters who were unaware of the experiment.

### mNSS

The mNSS is a scale used to assess the sensory, motor, reflex, and balance capabilities of mice. It provides a comprehensive evaluation with scores ranging from 0 to 18 (where 0 represents normal function and 18 indicates maximum impairment). Higher scores suggest poorer neurologic function.

### Rotarod test

The motor coordination and balance of mice were assessed using an accelerated rotating rod (KW-6C, Kew Basis, China). The mice were placed on the rotating bar, which gradually increased its speed from 4 to 40 rpm over a period of 300 s. To ensure accurate data collection, the tests were conducted 3 times daily with a half-hour break in between for the mice to fully recover and stabilize. We recorded the time it took for each mouse to be dislodged from the machine. The data used were the average of the 3 tests.

### Grip strength test

The grip strength test evaluates the animal’s capacity to exert its utmost force during gripping. The unrestrained left front paw automatically grips the rod of the grip strength meter when it comes into contact with the rod, then the mouse is gently pulled away from the device and the tensiometer displays the value of the pulling force. The measurements were repeated 3 times each.

### Gait analysis

The Gait Analysis System (TreadScan) is a video-based gait analysis for assessing gait and locomotion in mice. Each mouse walked freely in a clear, rolling corridor. A mouse’s steady movement across the rolling corridor without stopping and licking its fur was defined as a passable run. A camera was used to collect 20 consecutive seconds of 4 paw movements at a rate of 100 frames/s. Running average speed was then analyzed using TreadScan software (v4.00). Average speed was defined as the distance of the corridor divided by the time it took to cross the corridor.

### Measurements of brain infarct volume

On the 7th day post-dMCAO, the brain tissues were completely excised and cryopreserved at −20 °C for 30 min. Subsequently, the frozen brain tissues were sectioned coronally into approximately 1.5-mm-thick slices. These sections were then immersed in a staining solution containing 2,3,5-trityl tetrazolium chloride (TTC, T8877) from Sigma (USA). Following that, they were collectively processed at 37 °C within a warm box for a duration of 20 min. Finally, the brain tissue sections were fixed using paraformaldehyde (PFA) with a concentration of 4% obtained from China (C104188), and images of these sections were captured using a digital camera. The quantification of the infarct area was performed utilizing ImageJ software developed by the National Institutes of Health in Bethesda, USA [67]. To calculate the cerebral infarct volume as a percentage relative to hemispheric lesion volume after correcting for edema effects, we employed this formula: [total infarct volume − (ipsilateral hemisphere volume − contralateral hemisphere volume)] divided by contralateral hemisphere volume multiplied by 100% [67].

### Brain atrophy and tissue loss measurement

To assess brain atrophy volume, stroke mice were killed on day 28 after surgery. Fixation was performed after transcardiac perfusion with 4% PFA, and then brain tissue was taken out. Images of the entire brain were taken with a microscopic digital camera (AxioCam, Zeiss). The ImageJ system was utilized to measure the maximum width of the cortex, extending from the midline of the brain to the lateral edge of both the left and right hemispheres. Subsequently, a CWI was defined as the ratio of right (ischemic ipsilateral)/left (ischemic contralateral) cortical width × 100% [68].

### RNA-seq of brain tissue

Four mice in each group were killed on day 14 after stroke, and penumbral brain tissues were rapidly taken out and stored in a −80 °C refrigerator. The brain tissue samples were then sent to a Chinese company, Wuhan Seq Health Tech. Co., Ltd., located in Wuhan, for RNA-seq. Following the extraction of RNA from the samples, they underwent sequencing to identify any differential genes between the dMCAO and LIPUS groups. Subsequently, functional analysis using Gene Ontology (GO) was conducted to determine the functions of these differential genes in both groups. Additionally, pathway analysis using KEGG was performed to identify the pathways associated with myelin regeneration.

### BrdU injection

We first dissolved BrdU (B8010, Solarbio, China) in saline and configured it into a 10 mg/ml solution. Intraperitoneal injections were started on postoperative day 1 at a dose of 50 mg/kg/day for 14 days. Samples were taken on day 14 after dMCAO and the rate of cellular proliferation was evaluated by quantifying the count of cells labeled with BrdU.

### Immunofluorescent staining and analysis

After ensuring that the mice were under anesthesia, we first infused 0.9% saline through the hearts of the mice to flush out the blood and then infused 4% PFA to fix and remove the brain tissue. The removed brain tissue samples were then immersed in 30% sucrose. The samples were retrieved when they settled at the bottom and stored in a −80 °C refrigerator. Brain tissues were coronally sectioned using a cryosectioner (Thermo Fisher Scientific, USA) with a thickness of 15 μm per slice. The brain tissue sections were first exposed to 0.5% Triton X-100 for approximately 15 to 20 min at ambient temperature, followed by a subsequent incubation in the presence of 10% donkey serum for 1 h at a temperature of 37 °C. The samples were subsequently subjected to overnight incubation at a temperature of 4 °C with the following primary antibodies: mouse anti-non-phosphorylated neurofilament H (SMI-32, 1:100, 801701, BioLegend, USA), rabbit anti-myelin basic protein (MBP, 1:400, ab218011, Abcam, UK), mouse anti-NF200 (1:100, BM0100, Boster, China), rabbit anti-NeuN (1:500, A11954-1, Boster, China), mouse anti-PDGFRα(1:50, sc-398206, Santa Cruz Biotechnology, USA), sheep anti-BrdU (1:200, ab1893, Abcam, UK), rabbit anti-NG2 (1:200, ab275024, Abcam, UK), mouse anti-APC (1:100, OP80, Millipore, USA), and rabbit anti-Notch1 (1:100, 4380, Cell Signaling Technology, USA). The following day, the sections underwent a PBS wash and were subsequently incubated with their respective secondary antibodies at 37 °C for 1 h. Secondary antibodies used included the following: donkey anti-mouse 488(1:1,000, 715-545-150, Jackson ImmunoResearch, USA), donkey anti-sheep 594 (1:800, 713-585-003, Jackson ImmunoResearch, USA), donkey anti-rabbit 594 (1:800, 711-585-152, Jackson ImmunoResearch, USA), and donkey anti-rabbit 488 (1:1,000, 111-545-003, Jackson ImmunoResearch, USA). Finally, a small quantity of DAPI Fluoromount-G (0100-20, Southern Biotech, AL, USA) containing 4′,6-diamidino-2-phenylindole was introduced to the sections to stain the nuclei. Subsequently, immunofluorescence images were captured within a day using a Carl Zeiss LSM880 laser scanning confocal microscope. At least 3 microscopic fields of view around the lesion were captured for each section. Each mouse was sectioned at the anterior, middle, and posterior sites of the infarct lesion. The data analysis was conducted by an investigator who had no knowledge of the experimental design.

For cell death analysis, we performed TUNEL staining using a commercial kit (Superbrilliant AF594 TUNEL Apoptosis Kit, ZS-C31011, ZHONGSHI TONTRU) according to the manufacturer’s protocols.

During BrdU staining, brain sections were initially treated with 2 N HCl at 37 °C for a duration of 30 min to denature a portion of the double-stranded DNA. Subsequently, they were subjected to 3 consecutive washes with 0.1 mol/l boric acid (pH 8.5) for a duration of 5 min each time. Following sealing with donkey serum, the primary antibody was introduced and incubated overnight at a temperature of 4 °C, while the corresponding secondary antibody was employed on the subsequent day.

### Western blot

Proteins were obtained from brain tissue in the ischemic penumbra through the utilization of a kit for total protein extraction (p1250, Applygen, China). The BCA protein assay kit (23227, Thermo Fisher Scientific, USA) was utilized to determine the concentration of proteins extracted. The protein samples (30 μg each) were subjected to sodium dodecyl sulfate–polyacrylamide gel electrophoresis, followed by their transfer onto a polyvinylidene fluoride membrane (03010040001, Millipore, USA). The membrane was obstructed using 5% skim milk (BS102, Biosharp, China) for 1 h at ambient temperature. Subsequently, the primary antibody was added and incubated overnight at 4 °C, followed by the addition of a secondary antibody for 1 h at room temperature. The infrared imaging system from LI-COR Biosciences (USA) was utilized for detection, and the quantification of results was performed using ImageJ software. Primary antibodies employed were as follows: rabbit anti-β-actin (1:10,000, AB0035, Abways, China), rabbit anti-MBP (1:1,000, ab218011, Abcam, UK), rabbit anti-APC (1:2,000, ab40778, Abcam, UK), rabbit anti-IL-17A (1:500, A00421-2, Boster, China), rabbit anti-Notch1 (1:1,000,4380,Cell Signaling Technology, USA), and rabbit anti-cleaved Notch1 (1:1,000,4147, Cell Signaling Technology, USA). Dylight 800 Goat Anti-Rabbit secondary antibody (1:10,000, A23920, Abbkine, USA) was used in this study.

### Quantitative real-time PCR

All RNA from ischemic penumbra brain tissue was first taken according to the procedure of the Total RNA Rapid Extraction Kit (GK3016, Generay Biotech, China), and its concentration was subsequently quantified. In the second step, we synthesized cDNA by reverse transcription of equal amounts of RNA using the All-in-One-First-Strand cDNA Synthesis Kit (QP006, Gene Copoeia, China). Finally, we mixed cDNA with reagents from the All-in-One qPCR Mix kit (QP001, Gene Copoeia, China) and completed real-time fluorescence quantitative PCR on a LightCycler 480 (Roche Life Science, Switzerland). The primer sequences utilized are displayed in Table.

**Table.** Primers for RT-qPCR

Primers	Forward (5′–3′)	Reverse (5′–3′)
*Notch1*	CCAGTACAACCCACTACGGC	FTTGGAGAGGCTGCTGTGTAG
*Olig2*	CATGCACGACCTCAACATCG	TGAGGATGTAGTTTCGCGCC
*Sox10*	CCCAGATCGCCTACACTTCC	GGCTGAATAGAGGCCAGAGG
*Bcas1*	CACAGAGAACAGCAGCTCCA	CCTTCGTGTCTTCTGGGTCC
*Plp*	GCTGCCACTTACAACTTCGC	TGTGTGGTTAGCGCCTTGCT
*Mog*	GGTATCCCATCCGGGCTTTA	GGTGCTTGCTCTGCATCTTG
*CNPase*	AGATGAGGACACGGTGGCTA	GTAAGCATCAGCGGACACCA
*Mbp*	CTCAGAGTCCGACGAGCTTC	TCGCTGTGAGGGTCTCTTCT

### ELISA

Brain tissue homogenates were extracted according to the reagent instructions, and the concentration of IL-17A measured in the brain tissue homogenates was detected using a mouse IL-17A ELISA kit (RK00039, ABclonal, China).

### Statistical analysis

GraphPad Prism software (version 9.0.0, La Jolla, CA) was utilized for conducting statistical analysis. Before analysis, normality and variance chi-square tests were conducted on the entire dataset. For data that follow a normal distribution, the results are presented as the mean ± standard deviation. For data that do not follow a normal distribution, the results are presented as the median and interquartile range. To compare 2 groups with normally distributed continuous variables, an unpaired 2-tailed Student’s *t* test was employed. The Mann–Whitney *U* rank-sum test was used for nonnormally distributed continuous and discrete variables. When comparing means among multiple groups, we initially conducted one-way or 2-way analysis of variance (ANOVA) followed by Tukey’s multiple comparison test for pairwise comparisons. Repeated-measures ANOVA (neurobehavioral tests) was utilized to analyze differences in means over time between groups. In cases where significant variance was observed through ANOVA, a post hoc Bonferroni multiple comparison test was performed for all pairwise comparisons between conditions.

## Data Availability

The datasets used and/or analyzed in the current study are available from the corresponding author on reasonable request.
